# Rehabilitation Is a Global Health Priority

**DOI:** 10.1186/s12913-020-4962-8

**Published:** 2020-02-26

**Authors:** Allen W. Heinemann, Michael Feuerstein, Walter R. Frontera, Steven A. Gard, Leonard A. Kaminsky, Stefano Negrini, Lorie Gage Richards, Catherine Vallée

**Affiliations:** 1 Center for Rehabilitation Outcomes Research, Department of Physical Medicine and Rehabilitation, Feinberg School of Medicine, Northwestern University, Shirley Ryan AbilityLab, 355 E. Erie St Suite 14S, Chicago, IL 60611 USA; 2 ShirleyRyan AbilityLab, Chicago, IL USA; 3 Journal of Occupational Rehabilitation, Cary, NC USA; 4 Journal of Cancer Survivorship, Cary, NC USA; 5 Department of Physical Medicine, Rehabilitation, and Sports Medicine, University of Puerto Rico School of Medicine, San Juan, PR USA; 6 Department of Physiology and Biophysics, University of Puerto Rico School of Medicine, San Juan, PR USA; 7 American Journal of Physical Medicine and Rehabilitation, Cary, NC USA; 8 Department of Physical Medicine & Rehabilitation, Northwestern University, Chicago, IL USA; 9 Department of Veterans Affairs, Jesse Brown VAMC, Chicago, IL USA; 10 John & Janice Fisher Distinguished Professor of Wellness, Ball State University, Muncie, IN USA; 11 Department of Clinical and Experimental Sciences, University of Brescia, Brescia, Italy; 12 IRCCS Fondazione Don Gnocchi, Milan, Italy; 13 Department of Occupational and Recreational Therapies, University of Utah, Salt Lake City, UT USA; 14 Rehabilitation Department, Faculty of Medicine, Universite´ Laval, Quebec, Canada

## Rehabilitation Is a Global Health Priority

**Fig. 1:**
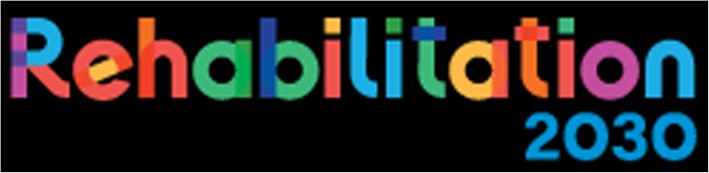
Logo for Rehabilitation 2030

The World Health Organization (WHO) launched an initiative in 2017 to promote universal access to rehabilitation when it hosted “Rehabilitation 2030: A Call for Action” [1] (Fig. 1). Attended by more than 200 rehabilitation experts from 46 countries, this meeting highlighted the unmet need for rehabilitation services and called for coordinated action and joint commitments by all stakeholders to raise the profile of rehabilitation. WHO and its partners committed to improving rehabilitation management and investment, building a high-quality rehabilitation workforce and services, and enhancing data collection.

The WHO convened a Second Global Rehabilitation 2030 Meeting in July 2019 that brought together stakeholders, including member states, international and professional organizations, nongovernment organizations, rehabilitation service users, and rehabilitation experts— including journal editors. The first 2 days included presentations on the current state of affairs of rehabilitation services in various countries and efforts made by several member states to integrate rehabilitation into their health care systems. Further, participants discussed strategies to make rehabilitation a political priority.

Over the next 2 days, the WHO’s staff, together with the Alliance for Health Policy and Systems Research, initiated a planning process for a health policy and research agenda related to rehabilitation. The objectives of this companion meeting were to (1) agree on a health policy and systems research framework for rehabilitation; (2) identify initial research questions; and (3) delineate enablers and barriers to building health systems and policy research capacity in rehabilitation.

Alarcos Cieza, PhD, the WHO’s Coordinator for Blindness and Deafness Prevention, and Disability and Rehabilitation, wrote in an article titled, “Rehabilitation the Health Strategy of the 21st Century, Really?” [2] that rehabilitation stakeholders must adopt a unified message that emphasizes the importance of functioning in order for it to become a political priority worldwide. She argues that coordinated advocacy by rehabilitation professional groups, subspecialties, and users is required to achieve this goal.

Her message highlights the critical work undertaken by the WHO to enhance access to rehabilitation, particularly in low- and middle-income countries. Accessible and affordable rehabilitation services are critical for people with chronic health conditions to maintain or increase their independence, participate in their communities, improve their economic productivity, and enhance their quality of life.

The aging population not only represents a major challenge to high-income nations but to low- and middle-income nations as well. The comorbid increase in the prevalence of chronic conditions and the aging of the world’s population contribute to an increasing number of people who experience declines in functioning. It is now recognized that optimizing functioning at all ages is a major global public health goal. Recent estimates of the global effect of rehabilitation on persons with health conditions is estimated to be much higher than the 1 billion people in the World Report on Disability published in 2011 [3]. Rehabilitation is unique in its contribution to this public health agenda because of its focuses on optimizing function. In fact, Jesus et al. [4] reported that the need for physical medicine and rehabilitation services has been increasing significantly in per capita terms as is the percentage of total years lived with disability globally and across countries of varying income levels. These authors also highlighted that this growth was greater in lower-income countries where rehabilitation is underresourced, emphasizing the pressing needs in these countries.

It is now essential to include data on functioning in health information systems in addition to the typical morbidity and mortality outcomes. These data can help us make better-informed decisions for the increased demand of rehabilitation services to enhance function as well as assist in the planning of health care systems for future expansion into these types of services.

It is evident to us that rehabilitation must be integrated fully into a nation’s health system and be strengthened specifically at the primary care level in order to increase access and achieve its full potential. As the WHO highlighted, we agree that health systems must be strengthened to assure that everyone who needs rehabilitation receives it. Equity should be a fundamental goal regardless of one’s social, economic, demographic, or geographic situation. WHO member states must also find solutions to the paucity of trained rehabilitation professionals and mechanisms to pay for the implementation of such services.

As editors-in-chief of rehabilitation journals, we unanimously accepted the invitation to participate in WHO’s Rehabilitation 2030 meetings and we embrace the concept of function as WHO’s third health indicator [5] along with mortality and morbidity. We recognize the increasing importance of health policy planning in improving access to rehabilitation services. In addition, we recognize that health policy requires a foundation of evidence on which health policy planning can build cost-effective systems and services. The emphasis of our journals varies widely and it is our diversity that supports the accumulating evidence base on which health policy planners, rehabilitation providers, users of rehabilitation services, and other stakeholders depend. We encourage authors to consider the global health policy implications of their research when they prepare their research reports for publication and to make these implications explicit. Together, we can fulfill a responsibility to enhance population health including enhanced function.

**--**

In order to encourage its wide dissemination this article is freely accessible on the following journal websites: *Archives of Physical Medicine and Rehabilitation, Journal of Cancer Survivorship, American Journal of Physical Medicine and Rehabilitation, Journal of Prosthetics and Orthotics, Journal of Cardiopulmonary Rehabilitation and Prevention, European Journal of Physical and Rehabilitation Medicine, The American Journal of Occupational Therapy, Canadian Journal of Occupational Therapy, BMC Health Services Research.*

--

## Data Availability

Not applicable.
